# Rise and Fall, and Rise Again: Phagosome Maturation Is Controlled by Two Kinases and One Phosphatase

**DOI:** 10.1371/journal.pbio.1001246

**Published:** 2012-01-17

**Authors:** Richard Robinson

**Affiliations:** Freelance Science Writer, Sherborn, Massachusetts, United States of America

**Figure pbio-1001246-g001:**
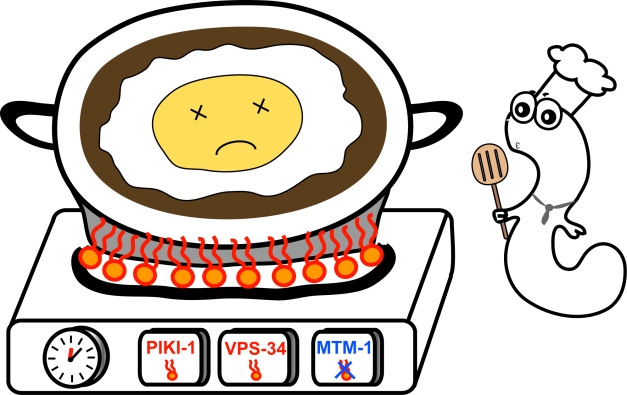
The precise temporal control of PtdIns(3)P (illustrated as flames) production and elimination on a phagosome (pot) by two kinases and one phosphatase (three controllers) is essential for the step-wise degradation of an apoptotic cell (fried egg) during *C. elegans* development.

[Fig pbio-1001246-g001]“Creative destruction” underlies not only dynamic economies, but also the development of multicellular organisms. After apoptosis, the programmed cell death that helps sculpt organs, dead cells are engulfed and degraded, by either surrounding cells or specialized phagocytes, such as the macrophages of the mammalian immune system.

Apoptotic cells are engulfed into a membrane-bound organelle called a phagosome. Engulfment triggers the production of a signaling molecule on the phagosome surface, called phosphatidylinositol 3-phosphate, or PtdIns(3)P. This molecule then attracts a suite of proteins that help bring digestive organelles, such as lysosomes and endosomes, to phagosomes in order to complete the degradation of the dead cell.

For reasons still unknown, the initial wave of PtdIns(3)P subsides after about 15 minutes, to be followed about 10 minutes later by a second, smaller wave. While the why remains obscure, the how is now much clearer, thanks to detailed work by Nan Lu, Zheng Zhou, and colleagues, who show that in *Caenorhabditis elegans*, the PtdIns(3)P oscillation is due to the coordinated efforts of two kinases, which create the signaling molecule, and one phosphatase, which destroys it. The authors show that the tight coupling of their opposed actions is required for successful degradation of the engulfed cell.

Previous work had identified that the so-called class III phosphatidylinositol 3-kinase (PI 3-kinase) VPS-34 was a major source of PtdIns(3)P on the endosome and phagosome surface. But apparently not the only source, since loss of VPS-34 diminished, but did not completely abolish, production of PtdIns(3)P, and produced an obvious, but not complete, loss of cell degradation, as evidenced by the accumulation of “cell corpses” within the engulfing cells.

The class II PI 3-kinases have been previously shown to produce PtdIns(3)P in vivo in response to both extracellular and intracellular stimuli, and the authors suspected that the worm's only class II PI 3-kinase, called PIKI-1, might be providing the residual signal. They showed that deletion of PIKI-1 also reduced the level of PtdIns(3)P, but again, not completely. Deleting the two together, however, completely abolished PtdIns(3)P on phagosomal surfaces, and halted phagosome maturation entirely. Without PtdIns(3)P, phagosomes did not accumulate one group of proteins called sorting nexins, or another group called RAB GTPases, which facilitate fusion of the phagosome with the digestive organelles.

But why use two kinases to accomplish a single purpose? PtdIns(3)P can be visualized in vivo with a fluorescent tag. By carefully tracking the timing of fluorescence, the authors found that with PIKI-1 but without VPS-34, PtdIns(3)P appeared rapidly, as normal, but then disappeared too quickly, and the second wave of PtdIns(3)P never materialized at all. Conversely, without PIKI-1 but with VPS-34, PtdIns(3)P accumulated slowly, but then its concentration fell and rose again more or less normally. Together, these results indicated that PIKI-1 was largely responsible for the initial burst of PtdIns(3)P production, while VSP-34 was needed for sustained expression in the first wave, and for the entire signal of the second wave.

But the properly timed loss of PtdIns(3)P is just as important for phagosome maturation, the authors found. Previously, the phosphatase MTM-1, which dephosphorylates PtdIns(3)P, was found to rescue the endocytosis defect of VPS-34 mutants, suggesting that it is involved in the regulation of endosomal trafficking. Here, when the authors knocked down MTM-1, cell corpses accumulated, indicating that phagosomes again could not progress to degradation. Remarkably, the effect of the loss of MTM-1 could be mitigated by reducing the amount of PIKI-1, indicating that it is the balance of actions of phosphorylation and dephosphorylation that is responsible for the degradation of cell corpses inside phagosomes.

The authors suggest that the oscillation in PtdIns(3)P may be required to facilitate binding multiple independent clusters of phagosome maturation factors, which must be brought into play in sequence. The identification of PIKI-1's role in production of PtdIns(3)P, and the working out of the temporal pattern of phosphorylation and dephosphorylation, should help lead to the rapid identification of these various factors. The results are not just significant for understanding roundworm biology, since mammals also have class II and III PtdIns(3)P kinases, and it is likely that a similar scheme for phagosome maturation operates in macrophages, the authors note.

**Lu N, Shen Q, Mahoney TR, Neukomm LJ, Wang Y, Zhou Z (2012) Two PI 3-Kinases and One PI 3-Phosphatase Together Establish the Cyclic Waves of Phagosomal PtdIns(3)P Critical for the Degradation of Apoptotic Cells. doi:10.1371/journal.pbio.1001245**

